# Postpartum Health in Mothers of Preterm Infants in the NICU: Needs, Service Utilization, and Care Gaps—A Systematic Review

**DOI:** 10.3390/healthcare14050668

**Published:** 2026-03-06

**Authors:** Tabea Mina Stein, Marie-Jeannine Riefert, Harald Abele, Cornelia Wiechers, Claudia F. Plappert

**Affiliations:** 1 Obstetrics and Gynecology, University Hospital Tübingen, 72076 Tübingen, Germany; 2 Studies and Teaching Division, Faculty of Medicine, University Tübingen, 72076 Tübingen, Germany; 3 Obstetrics and Perinatal Care, University Hospital Tübingen, 72076 Tübingen, Germany; 4 Midwifery Science, University Tübingen, 72076 Tübingen, Germany; claudia.plappert@med.uni-tuebingen.de; 5 Neonatology and Perinatal Care Centre, University Tübingen, 72076 Tübingen, Germany

**Keywords:** mother, preterm infant, preterm birth, neonatal intensive care unit NICU, health needs, physical needs, psychological needs, support needs, health behavior, coping

## Abstract

**Background/Objectives:** Mothers of preterm infants face complex postpartum challenges, yet their needs are often overlooked in neonatal care. This review synthesizes evidence on maternal health needs, service utilization, perceived adequacy, and barriers to care. **Methods:** A systematic search of PubMed, CINAHL, and PsycINFO identified 16 peer-reviewed studies published between 2007 and 2025 on mothers of preterm infants. A narrative synthesis integrated quantitative and qualitative findings from NICU settings. **Results:** Across 16 included studies, all reported psychological and emotional needs, with anxiety, distress, and fear for infant survival frequently highlighted. Informational needs were identified in 11 studies, particularly regarding infant care and postpartum guidance. Physical needs were reported in 10 studies, including fatigue and pain affecting NICU engagement. Nursing support and lactation services were consistently accessed, whereas psychosocial services and postpartum follow-up were reported in fewer than half of the studies. Perceived adequacy depended on empathetic, individualized communication, while structural and contextual barriers, such as transportation, financial burden, and NICU policies, limited maternal engagement. Despite available services, gaps in emotional, informational, and practical support persisted. **Conclusions:** Mothers of preterm infants experience substantial postpartum health needs that are insufficiently addressed within current NICU-centered care structures. Integrating maternal-focused, continuous, and psychosocially informed postpartum care into neonatal services is essential to reduce care gaps and support maternal well-being during NICU hospitalization.

## 1. Introduction

Mothers of preterm infants experience substantially higher levels of stress, particularly when their infants require care in a neonatal intensive care unit (NICU) [1,2]. In this context, maternal health and well-being are often overlooked, despite increased risk of physical complications, psychological distress, and unmet postpartum healthcare needs, which may be compounded by structural barriers to accessing care while remaining present at the NICU [3].

### 1.1. Preterm Birth and Maternal Stress

Preterm birth is a frequent obstetric event worldwide. Approximately 15 million premature births occurred worldwide in 2015 [4]. In Germany, the proportion of premature birth has remained relatively stable at around 8% for several years [5]. A preterm birth is defined as a delivery that occurs before the completion of 37 weeks of gestational age (GA). Prematurity can be conceptualized using two classification systems: gestational age is the official basis for subclassification into the following categories: extremely preterm (<28 weeks GA), very preterm (28–31 weeks GA) and moderate to late preterm (32–36 weeks GA) [6]. Birth weight is a separate classification dimension that distinguishes between low birth weight (LBW; <2500 g), very low birth weight (VLBW; <1500 g), and extremely low birth weight (ELBW; <1000 g) [7].

Beyond its epidemiological relevance, preterm birth constitutes a critical context for maternal well-being. Compared to mothers of term-born infants, mothers of preterm infants consistently report higher levels of stress, anxiety, and emotional distress, particularly during the early postpartum period [1,8]. The causes of this are multifaceted, encompassing the sudden and often unexpected transition from pregnancy to birth, increased medical interventions aimed to preventing preterm birth, and concerns about the infant’s health outcomes [9,10,11,12,13,14,15,16,17].

Prevention and management of threatened preterm birth often involve pharmacological and interventional measures that may impose additional physical and psychological burdens on mothers. Tocolytic therapy used to delay labor has been associated with cardiovascular side effects such as tachycardia and hypotension and, in rare cases, pulmonary complications [10]. Similarly, the prophylactic administration of glucocorticoids for fetal lung maturation can trigger short-term metabolic changes such as hyperglycemia or blood pressure fluctuations [11]. Invasive interventions such as cervical cerclage or the insertion of a cervical pessary are associated with risk of infection, bleeding, or pain and may further increase the physical strain on the mother [12]. Beyond their physical effects, these preventive interventions are frequently experienced as psychologically burdensome. The need for inpatient monitoring or repeated clinical check-ups can lead to stress, anxiety, and restrictions in everyday life, especially when there is uncertainty about the outcome of the pregnancy [13]. Repeated medication intake or monitoring can also increase the subjective perception of stress and impair the mother’s quality of life [14].

If preterm birth cannot be prevented, the mode and place of delivery significantly influence infant outcomes and indirectly affect maternal postpartum experiences [15,16,17,18]. In practice, mothers of preterm infants are more likely to give birth via cesarean section, particularly in cases of early gestational age or breech presentation [15,18].

The place of birth is also crucial. Women at risk of preterm birth should ideally give birth in tertiary centers with NICUs. These centers not only offer optional neonatal care but also ensure specialized obstetric care for the mother [16]. Birth in lower-level facilities without immediate access to surgical and intensive care resources are associated with higher maternal morbidity and delayed necessary interventions [17].

Following preterm birth, a substantial proportion of infants require hospitalization in a NICU, with admission rates and length of stay increasing as gestational age decreases [19,20]. NICU hospitalization exposes mothers to a highly medicalized and emotionally demanding environment, characterized by continuous monitoring of the infant, uncertainty about health outcomes, and rapid adaptation to complex clinical routines [1,3].

Prolonged neonatal hospitalization has important consequences for mothers and families. Mothers are commonly accommodated near their infants, for example, affiliated family housing, to facilitate bonding, breastfeeding, and parental involvement in care. Family-centered and family-integrated care models during extended NICU stays have been shown to improve parental well-being, participation in care, and mother-infant interaction [21,22]. In addition to the close proximity of the accommodation, mothers of preterm infants admitted to a NICU are to be supported by multidisciplinary psychosocial care structures. Psychosocial support is considered an integral component of neonatal intensive care. Mothers are supported by specially trained nursing staff, psychologists or psychosocial professionals, social workers, and, where needed, pastoral or spiritual care services. Core elements of care include early and continuous parental involvement, structured information and communication support for bonding and breastfeeding, and screening for psychological distress. In Germany, these support structures are typically embedded within perinatal presence [21].

### 1.2. Postpartum Period

The postpartum period, or puerperium, refers to the time following childbirth and generally extends for six to eight weeks. It is commonly divided into an early phase (first ten days) and a late phase (day eleven to the end of week twelve) [23]. During this period, the maternal body undergoes profound physiological adjustments to recover from pregnancy and childbirth. Following placental delivery, estrogen and progesterone levels rapidly decline, while prolactin secretion increases to initiate and maintain lactation. Uterine involution occurs over approximately six weeks, accompanied by the gradual transition of lochia from bloody to serous and finally whitish discharge. Additional physiological changes include normalization of blood volume and cardiovascular function, as well as recovery of pelvic floor and abdominal musculature [23,24].

Alongside these somatic processes, the puerperium represents a critical period for psychological adaptation and the development of maternal identity. Hormonal fluctuations, physical recovery, and sleep deprivation contribute to emotional variability, particularly during the early postpartum phase [25]. Transient mood disturbances are common and typically resolve spontaneously within the first days after childbirth. Beyond this initial phase, the postpartum period is essential for establishing maternal-infant bonding and the parent–child relationship [23,26].

Compared to the typical postpartum course after term birth, the postpartum period after a premature birth has both physiological and psychological characteristics. Physically, the recovery process can generally proceed normally, but postpartum recovery is often affected by medical complications, operative deliveries, and difficulties establishing lactation. In particular, the lack of or limited direct breastfeeding and the need to pump milk can alter hormonal regulation and the physical experience of the postpartum period [27,28].

The NICU context can disrupt key processes of early motherhood and may further contribute to maternal distress during the postpartum period. Psychologically, preterm birth has been consistently associated with higher levels of maternal distress, including increased symptoms of anxiety, depression, and stress, as well as a high prevalence of posttraumatic stress symptoms in the early postpartum period. And studies indicate that psychological distress related to preterm birth can persist well beyond the initial postpartum phase, with many women showing elevated stress markers and trauma-related symptoms weeks to months after delivery [8]. These responses are shaped not only by the early and unexpected timing of childbirth but also by the environmental and organizational context of neonatal intensive care. Mothers of preterm infants frequently face prolonged separation from their infants, disrupted opportunities for early mother-infant contact, and the stress of navigating the technical and emotionally intense NICU environment. Quantitative research demonstrates that maternal stress related to the NICU is significantly elevated compared with normative postpartum experiences [1].

### 1.3. Postnatal Care

Internationally, the World Health Organization (WHO) defines postnatal care as a critical component of maternal and newborn health. The WHO recommends early and repeated postnatal contacts to address physical recovery, mental health, psychosocial well-being, and the prevention of complications during the weeks following childbirth. These recommendations emphasize the importance of continuous, accessible, and mother-centered care throughout the postpartum period [29].

In Germany care is delivered in two main settings: in-hospital immediately after birth, and home-based care provided by midwives according to statutory health insurance regulations (§§ 24c, 24d, § 134a German Social Code Book [SGB] V). Hospital stays typically last around 3.5 days, during which monitoring, breastfeeding support, wound checks, and newborn examinations are provided [30,31].

Home based midwifery care begins immediately after discharge and can continue for up to twelve weeks postpartum, including visits to support maternal recovery, breastfeeding, parenting skills, and mother-infant bonding. Part of the care also includes a postnatal check-up with the gynecologist, which usually takes place about six weeks after the birth [32].

### 1.4. Maternal Service Utilization and Gaps

Even though all insured women in Germany are legally entitled to midwifery care during the postpartum period, there are substantial differences in the actual utilization of these services. Socioeconomic factors play a significant role: analyses of BARMER routine data indicate that mothers with lower income, despite having the same legal entitlement, are less likely to use midwifery services. Educational level and lack of awareness that midwifery care is fully covered by statutory health insurance also negatively affect utilization [33]. Regional shortages of midwives can result in gaps in service provision [34]. Early contact with a midwife during pregnancy increases the likelihood of receiving postpartum home visits [33]. Additionally, women in psychosocially vulnerable situations, such as those with higher needs or previous negative experiences with healthcare accessibility, may face additional barriers [35]. Subjective factors, including trust in the midwife and perceived practical support, further influence utilization, while cultural or language barriers may additionally reduce the likelihood of engaging midwifery care [35,36].

Based on these findings and the known risk factors for preterm birth, one might initially assume that mothers of preterm infants would make less use of midwife-led postnatal care. This is compounded by the fact that the pregnancy is abruptly shortened and the search for a midwife may not have been initiated or completed at the time of delivery [37,38]. In addition, preterm births are often associated with longer prenatal hospital stays [39], which can limit participation in individual or group-based birth preparation. As a result, affected women may enter labor less prepared, even though active engagement with birth preparation positively affects both labor outcomes and postpartum psychological health [40,41].

In some cases, access to organized midwifery care may be limited because the responsible midwife is unable to travel the required distance to provide care while the infant is being treated at a specialized perinatal center. Although these factors collectively point to a potential undersupply, analyses of routine data show no significant difference in the number of postnatal midwife visits between mothers of preterm and term infants. However, routine data capture only the frequency of visits and do not provide insight into the timing, content, or adequacy of care in relation to mothers’ complex needs [33].

When broader dimensions of care utilization are considered, this finding contrasts with international evidence, which consistently shows that mothers of infants hospitalized in NICUs often neglect their own health or underutilize maternal health services. For example, a high proportion of mothers do not attend postpartum care appointments or attend them late after NICU stays. In a US mixed-method study, NICU mothers reported postponing medical care for themselves to remain at their child’s bedside, and stakeholders identified systemic barriers such as unclear responsibilities and reimbursement issues as obstacles to maternal health care utilization [3]. Another study indicates that in cases of transfer or relocation, mothers were even less likely to attend postpartum check-ups within six weeks, even if they had existing health complaints [3,42].

From a health services research perspective, healthcare service utilization cannot be understood as a direct automatic response to health needs alone. Established theoretical approaches emphasize that service use emerges from the interaction of individual, social, and structural determinants. Andersen’s behavioral model of health service use conceptualizes utilization as the result of health needs, predisposing characteristics, enabling resources, and healthcare system factors [43]. Within this framework, individuals with substantial health needs may still underutilize available services if access is limited, services are poorly coordinated, or if they are not perceived as relevant.

The postpartum period is a particularly sensitive phase in which competing demands, physical recovery, and emotional strain may further influence help-seeking behavior [37,38]. For mothers of preterm infants who require hospitalization in the NICU, utilization of maternal health services may additionally be shaped by contextual factors such as spatial separation from the infant, time constraints, and prioritization of infant-focused care [3].

Consequently, service utilization patterns in this group may reflect not only individual preferences or needs, but also structural conditions that constrain access to and engagement with postpartum care. As a result, it remains unclear how well current care structures align with the needs of mothers of hospitalized preterm infants and where gaps in postpartum care persist. Despite growing evidence on the psychological burden associated with preterm birth and NICU hospitalization, existing research has largely focused on neonatal outcomes or maternal mental health in isolation. Less attention has been paid to the broader spectrum of postpartum health needs of mothers whose infants remain hospitalized in NICUs, particularly regarding their utilization of health and support services during this period.

### 1.5. Aim

To better understand the alignment between maternal postpartum health needs and existing care structures during NICU hospitalization, this study aims to examine how postpartum health needs of mothers of preterm infants (<37 weeks’ GA) are addressed during hospitalization in NICUs including service utilization and perceived care gaps. Accordingly, the following research questions are addressed:

1. What are the physical, psychological, emotional, and informational postpartum health needs of mothers during NICU hospitalization?
2. Which health and support services are offered and utilized, and to what extent do they meet mothers’ needs?
3. What barriers or gaps exist between maternal needs and the available postpartum care?

## 2. Materials and Methods

This systematic review was conducted and reported in accordance with the PRISMA 2020 guidelines to ensure transparency, completeness, and reproducibility. It examined the postpartum health needs of mothers of preterm infants (<37 weeks’ gestational age) during hospitalization in neonatal intensive care units (NICUs), including patterns of health and support service utilization and perceived gaps in care [44].

The review followed established systematic review methods to ensure transparency and reproducibility. Due to substantial heterogeneity in study designs, population, outcome measures, and methodological approaches, a meta-analysis was not feasible; therefore, a narrative synthesis was conducted. The protocol has been registered with PROSPERO (ID: CRD420251246203).

### 2.1. Eligibility Criteria

Eligibility criteria were defined to include diverse study designs and outcome domains, reflecting the complexity and multidimensional nature of postpartum health and healthcare utilization in this population. Studies were eligible if they included mothers of preterm infants during NICU hospitalization and reported on postpartum health needs, utilization of health or support services, perceived support, barriers, or coping strategies. Studies were eligible if they reported on at least one of the listed maternal postpartum needs (physical, psychological, emotional, informational), rather than requiring all domains to be addressed. Eligible design comprised qualitative studies (interviews, focus group, ethnographic), quantitative observational studies (surveys, needs assessments, questionnaires), and mixed-methods studies. Studies focusing solely on fathers, other family members, healthcare staff, infants’ outcomes without maternal relevance, interventional studies, editorials, commentaries, systematic reviews, conferences abstracts, academic dissertations/theses, or grey literature were excluded. No language or date restrictions were applied (Table 1).

### 2.2. Search Strategy

The literature search was conducted between the 20 November and 10 December 2025 and covered publications from database inception to the search date. The following electronic databases were searched: MEDLINE (via PubMed), CINHAL, APA PsycINFO, and Web of Science.

Search strategies combined controlled vocabulary (MeSH, Emtree, CINAHL Headings, APA Thesaurus) and free-text terms for mothers, preterm infants, NICU, health needs, service utilization, and coping: (mother* OR maternal OR “maternal health”) AND (“preterm infant*” OR “premature neonate*” OR “NICU”) AND (health need* OR support* OR psychological need* OR coping* OR “health behavior” OR utilization) AND (postpartum OR postnatal). The complete search strategies and results for each database are presented in Table 2.

No language restrictions were applied. Titles and abstracts of non-English, non-German or non-Italian records were initially screened using automated translation tools. Where full-text articles were considered potentially eligible, full texts were translated using translation software (DeepL Translator, https://www.deepl.com, accessed on 15 February 2026), and key sections (methods and results) were reviewed to ensure conceptual accuracy and consistency during eligibility assessment and data extraction.

Additionally, studies were screened via forward citation searching, and reference list of included studies were checked systematically. The search strategy was intentionally broad in order to capture the full range of postpartum health needs, service utilization patterns, and care experiences of mothers with preterm infants in NICU settings.

### 2.3. Study Selection and Data Extraction

This study selection process is reported in accordance with the PRISMA 2020 guidelines and illustrated using a PRISMA flow diagram [44]. All records retrieved from the database searches were imported into EndNote 21, and duplicates were removed prior to screening. Titles and abstracts were screened for eligibility followed by full-text-assessment, by a single reviewer. Eligibility criteria were defined a priori and applied consistently throughout the screening process. Reasons for exclusion at the full-text stage were documented.

Data extraction was performed by a single reviewer using a standardized data extraction form. Extracted data included study characteristics (country, setting, design), sample characteristics, maternal postpartum health needs, utilization of health and support services, perceived support, barriers, coping strategies, and psychosocial outcomes.

Full text articles that could not be accessed despite repeated attempts (*n* = 12) were documented and are reported in the PRISMA flow diagram. These studies could not be included in the review due to limited availability in online databases or institutional subscriptions. The inability to access this full text may represent a limitation in the completeness of the evidence based and could potentially introduce selection bias. Efforts to identify studies through citation tracking and reference list screening were undertaken to mitigate the risk.

To further enhance transparency and reproducibility, all inclusion and exclusion decisions were documented in a structured screening log.

### 2.4. Outcomes

Primary outcomes include maternal postpartum health needs (physical, psychological, emotional), utilization of health and support services, perceived fulfillment of needs, barriers and gaps in care, coping strategies, and psychosocial support, as reported through validated surveys, assessments, interviews, or observational measures during NICU hospitalization.

### 2.5. Data Synthesis

Findings were synthesized narratively and thematically, following the framework proposed by Popay et al. [45]. The narrative synthesis aimed to identify recurring themes, patterns, and gaps across studies rather than to quantify effects sizes.

**Figure 1:**
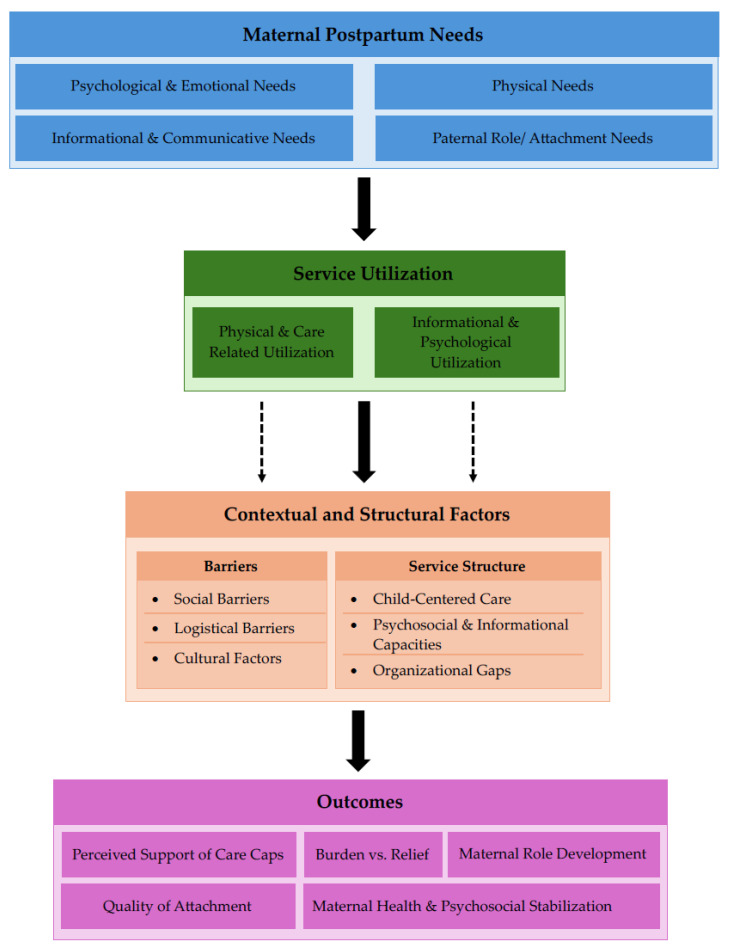
Preliminary theoretical framework of maternal postpartum needs and service utilization.

A preliminary theoretical framework (Figure 1) was developed a priori based on the review’s research questions and relevant literature to guide the narrative and thematic synthesis. Figure 1 provides a conceptual overview, illustrating the assumed relationships between maternal postpartum needs, utilization of healthcare and support services, contextual and structural factors, and perceived outcomes. The framework was used as a tool to organize and interpret findings but was not modified during the synthesis process.

First, a preliminary synthesis was conducted by grouping study findings thematically. Qualitative findings were coded inductively and organized into recurring themes, while quantitative results were summarized descriptively and mapped onto the same thematic structure. This process allowed convergence and complementarity between qualitative and quantitative evidence to be examined.

Second, the relationships within and between studies were explored by comparing findings across study designs, settings, and contexts. Particular attention was paid to how different needs were associated with service utilization patterns, perceived adequacy of care, and reported barriers. Finally, the robustness of the synthesis was considered by reflecting on methodological limitations of the included studies, variations in study quality, and the consistency of findings across diverse contexts.

### 2.6. Quality and Certainty Assessment

All included studies were retained in the review regardless of their methodological quality. Quality assessment was conducted to appraise the rigor and credibility of individual studies, and to inform the interpretation and discussion of findings. Studies were systematically evaluated using the CASP checklist for qualitative and quantitative designs, and the MMAT for mixed-methods [46,47]. Studies meeting ≥80% of criteria were considered high quality, 60–79% moderate, and <60% low.

For quantitative outcomes, the certainty of evidence was assessed using the GRADE approach, and for qualitative findings, the GRADE-CERQual was applied [48,49]. Different appraisal tools were applied to ensure methodological rigor across diverse study designs and to allow for a design-specific assessment of evidence quality. GRADE-CERQual assesses confidence in evidence based on methodological limitations, relevance, coherence, and adequacy of data, while GRADE for quantitative studies considers risk of bias, inconsistency, indirectness, imprecision, and publication bias.

The results of these assessments were used to contextualize the strength and reliability of the evidence when synthesizing findings, but no studies were excluded based on solely on quality appraisal. This approach allowed all available evidence to be considered while reflecting the methodological limitations and certainty of the included studies in the narrative synthesis.

## 3. Results

### 3.1. Study Selection

The database search identified a total of 1014 records (MEDLINE *n* = 482; CINAHL *n* = 137; APA PsycINFO *n* = 81; Web of Science *n* = 313) with one additional record identified through citation searching. After removal of 275 duplicates, 739 records remained for title and abstract screening. Of these, 696 records were excluded as they did not meet the inclusion criteria.

A total of 43 full-text articles were assessed for eligibility. Twelve full texts were not accessible despite repeated attempts to obtain them.

The remaining 31 accessible full texts were screened in detail, of which 15 were excluded for reasons such as wrong population [50,51,52], wrong setting (post discharge) [53,54,55,56,57], or lack of maternal outcome [58,59,60,61,62,63,64].

**Figure 2:**
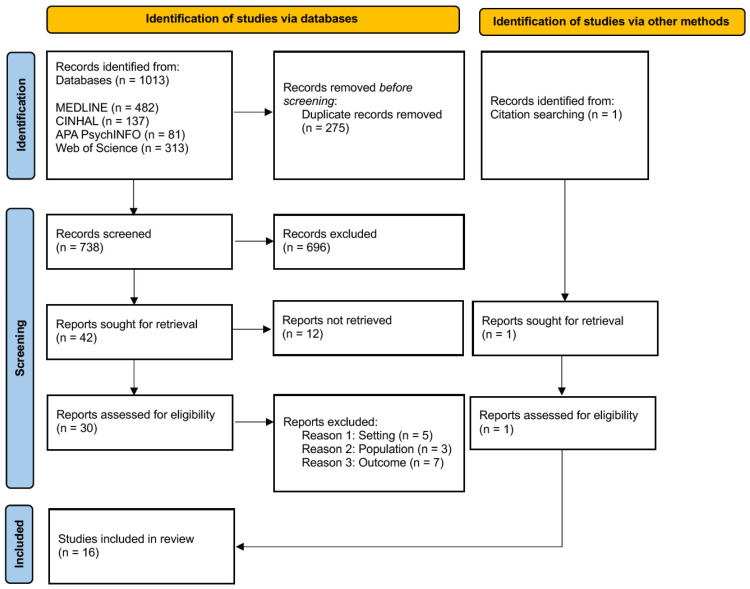
Identification, screening, and inclusion stages following PRISMA [47].

Ultimately, 16 studies met the inclusion criteria and were included in the final synthesis [65,66,67,68,69,70,71,72,73,74,75,76,77,78,79,80]. The study selection process is illustrated in the PRISMA flow diagram (Figure 2) [44].

### 3.2. Descriptive Characteristics of Included Studies

Sixteen studies published between 2007 and 2025 were included in this review. The studies were conducted across Africa (Ghana) [65,66,72], North America (United States [68,71,78,79], Canada [69]), Europe (Germany [76], France [75], Italy [74]), Asia (Saudi Arabia [67], Taiwan [70], South Korea [77]), and Oceania (Australia [73], New Zealand [80]).

Most studies employed qualitative designs (*n* = 11) [65,66,67,69,70,71,72,73,74,75,76,79], complemented by quantitative cross-sectional or longitudinal cohort studies (*n* = 4) [68,74,77,80] and one mixed-methods study [75]. Data collection primarily involved semi-structured interviews [66,67,69,70,72,73,75,76,78,79], focus groups [65], or validated self-report questionnaires [68,74,75,77,80].

All studies focused on mothers of preterm infants (<37 weeks’ gestation) cared for in neonatal intensive care settings. Sample sizes ranged from 5 [67] to 133 [80] mothers. Considerable heterogeneity was observed in gestational age and clinical risk, ranging from late preterm infants (32–36 weeks) to very preterm and very low birth weight infants (≤32 weeks or <1500 g). Timing of data collection varied from the acute NICU hospitalization period to post-discharge and longer-term follow-up.

Study settings included NICUs of varying acuity. Some studies were conducted in Level II NICUs [69,80], while the majority took place in Level III NICUs [65,66,68,74,76,77,79]. One study was conducted in a Level IV NICU [70], and a few studies included settings spanning Level III-IV NICUs [76,79]. Several studies did not specify the NICU level [67,70,73,75,78]. This range of NICU levels reflects variability in hospitals resources, intensity of neonatal care, and the clinical complexity of included infants.

Across studies, outcomes addressed maternal psychological and emotional needs [65,66,67,68,69,70,71,72,73,74,75,76,77,78,79,80], informational needs related to infant care and communication with healthcare professionals [65,66,67,69,70,71,74,75,76,77,78], and, to a lesser extent, physical health needs [66,70,71,73,77]. Several studies examined the use of formal and informal support services [65,66,67,68,69,71,72,73,75,76,78,79] and identified structural, socioeconomic, and cultural barriers to care [65,66,67,68,69,71,72,73,74,75,76,78,79]. Given the substantial methodological and clinical heterogeneity across studies, findings were synthesized narratively. Detailed study characteristics are presented in Appendix A in Table A1.

### 3.3. Quality Assessment/Risk of Bias

The methodological quality of the included studies was systematically assessed using the CASP checklists for qualitative and quantitative designs, and the MMAT for the single mixed-methods study. Studies meeting ≥80% of the criteria were considered high quality, 60–79% moderate, and <60% low. Of the included studies, 11 were qualitative, 4 quantitative, and 1 was a mixed-methods study. Most qualitative studies were rated as high quality, reflecting clear aims, appropriate designs, transparent data collection, and rigorous analysis [65,66,67,69,70,71,72,73,76,78,79]. Quantitative studies were generally moderate to high quality, with occasional limitations in sample size, reporting, or confounder control [68,74,77,80]. The single mixed-methods study [75] demonstrated high qualitative rigor and moderate quantitative quality; however, the lack of integration between qualitative and quantitative components resulted in an overall low mixed-methods rating. While no studies were excluded based on quality or risk of bias, limitations identified through CASP and MMAT assessments, such as small sample sizes, unclear recruitment strategies, or insufficient integration in mixed-methods designs, were considered when synthesizing findings. The final quality assessments, including overall ratings and risk of bias, are summarized in Table 3.

Most qualitative findings were supported by moderate to high confidence according to GRADE-CERQual, whereas quantitative evidence was limited and of low certainty. Detailed GRADE and GRADE-CERQual assessments are provided in Table A1.

No studies were excluded based on quality or risk of bias, but limitations identified through CASP and MMAT assessments, such as small sample sizes, unclear recruitment strategies, or insufficient integration in mixed-methods designs, were considered when synthesizing findings. The summary of certainty of evidence assessment can be found in the Appendix B under Table A2.

### 3.4. Narrative Synthesis

The findings are presented in alignment with the preliminary theoretical framework (Figure 1), reporting identified postpartum needs, received healthcare and support services, patterns of service utilization and perceived adequacy, contextual and structural influences, and reported gaps in care.

A condensed summary of study contributions is presented in the main text, with a detailed overview of all included studies provide in the Appendix C in Table A3.

#### 3.4.1. Identified Postpartum Needs of Mothers of Preterm Infants

Across the included studies, mothers of preterm infants described a broad and interrelated set of postpartum needs that extended beyond physical recovery and encompassed psychological, informational, and social dimensions. Needs within this domain were reported in all but one study and were consistently portrayed as overlapping rather than discrete, reflecting the complexity of the postpartum experience in the context of neonatal intensive care.

Psychological and emotional needs were the most frequently addressed and were identified in 16 of the 16 included studies [65,66,67,68,69,70,71,72,73,74,75,76,77,78,79,80]. Experiences of anxiety, emotional distress, fear for the infant’s survival, and feelings of helplessness were consistently reported. Several quantitative studies reported elevated depressive symptom scores or increased stress levels during the NICU stay. Emotional distress was closely linked to uncertainty regarding the infant’s prognosis, prolonged separation after birth, and a perceived loss of control over both the birth experience and the infant’s care. Mothers described a sustained state of emotional vigilance that often persisted beyond the immediate postpartum period, influencing how they interpreted information, interacted with healthcare professionals, and evaluated the care they received.

Informational needs were reported in 11 studies and were closely intertwined with emotional well-being [65,66,67,69,70,71,75,76,77,78,79]. Mothers expressed a strong desire for clear, consistent, and timely information regarding their infant’s medical condition, expected course, and care requirements, as well as guidance on their own postpartum recovery and caregiving role. A substantial proportion of mothers reported having received no structured postpartum education. Where information was fragmented, contradictory, or delivered without sensitivity to emotional readiness, mothers described confusion, uncertainty, and diminished confidence. Information deficits were therefore experienced not merely as a lack of knowledge but as a source of emotional strain and reduced agency.

Physical needs were addressed in 10 studies and were particularly salient in the early postpartum period [66,69,70,71,73,75,76,77,78,79]. Mothers described persistent fatigue, postoperative and musculoskeletal pain, and reduced physical functioning, often following operative delivery. Physical recovery was described not only as a medical process but as a determinant of mothers’ capacity to be present in the NICU, to tolerate prolonged bedsides stays, and to participate in caregiving activities such as holding, feeding, or expressing milk. Physical limitations therefore directly shaped maternal engagement and interaction with healthcare services.

Social and relational needs were identified in 15 studies and reflected mothers’ reliance on both professional and informal sources of support [65,66,67,68,69,70,71,72,73,74,75,76,77,78,79]. Emotional availability, reassurance, and continuity of care from health professionals, particularly nursing staff, were described as central to mothers’ ability to cope with the demands of the postpartum period. Support from partners and family members was also important, although its availability and form varied across cultural and contextual settings. Peer support from other parents with neonatal intensive care experience was reported less consistently but was described as uniquely validating when available, offering understanding that completed professional support.

#### 3.4.2. Healthcare and Support Services Received

The included studies described a range of healthcare and support services provided to mothers during the postpartum period and their infant’s NICU stay. These services varied in scope, organization, and degree of integration into routine care, and were not uniformly available across settings or study contexts.

Medical and nursing care constituted the core of postpartum and neonatal support and were reported in all studies. Nursing staff were consistently described as the primary point of contact for mothers, providing clinical care, explanations of procedures, and guidance in day-to-day caregiving tasks [65,66,67,68,69,70,71,72,73,74,75,76,77,78,79,80]. Beyond technical competence, the interpersonal aspects of nursing care, availability, continuity, and responsiveness, were repeatedly emphasized as shaping mothers’ experiences [65,69,70,72,76,79]. Nurses were often described as mediators between mothers and the wider healthcare system, particularly in highly medicalized NICU environments [65,69,70].

Information and education services were described in many studies and included explanations of the infant’s health status, instruction in caregiving activities, and guidance related to feeding and milk expression [65,66,67,69,70,71,74,75,78,79]. However, these services were not uniformly structured or delivered. Considerable variability was noted in when information was provided, how it was framed, and whether it was coordinated across professional roles. In some settings, information provision appeared fragmented, whereas in others it was described as more continuous and responsive to mothers’ evolving needs. Several studies reported that information focused primarily on the infant, with limited attention to mothers’ own postpartum recovery [65,69,70,74,78].

Psychological support services, such as counseling or psychological support, were reported less consistently and were often described as optional or dependent on referral rather than integrated into routine postpartum or neonatal care [73]. As a result, access to emotional support appeared uneven and influenced by institutional practices rather than systematically aligned with the level of psychological distress reported by mothers. Formal mental health screening or proactive referral pathways were rarely described [73,77].

Lactation and feeding represented a distinct area of service provision and was reported in a majority of studies [65,69,71,74,78]. Support related to milk expression and feeding was often intensive and initiated early in the postpartum period [65,69,74]. Mothers described frequent contact with healthcare professionals regarding pumping schedules, milk supply, and feeding progression [65,69,78], while this support was commonly valued as a means of contributing to the infant’s care, it was also associated with physical exhaustion and emotional pressure, particularly when milk production was positioned as a primary marker of maternal involvement [78]. This dual role of lactation support, as both supportive and burdensome, was evident across multiple study contexts [64,65,69,78].

Overall, the provision of healthcare and support services was characterized by a strong focus on neonatal clinical needs, with maternal support often embedded within infant-centered care rather than delivered as a distinct postpartum service.

#### 3.4.3. Service Utilization

Patterns of service utilization reflected both the availability of services and mothers’ capacity to engage with them. Utilization differed markedly between service types, with nursing support [65,67,69,72,73,76,78,79] and lactation-related services being accessed most frequently [65,69,71,74,78], whereas psychosocial services [73,76,77] and postpartum follow-up care were accessed less consistently [71,77].

Nursing support was described as continuously available during NICU hospitalization and was therefore the most frequently utilized form of support [65,67,69,72,73,76,78,79]. Mothers reported regular interactions with nursing staff related to infant care, feeding, and daily routines [65,69,72,76,78]. Because nursing care was embedded in routine NICU practice, its use did not generally require additional referral or active help-seeking by mothers [65,72,76]. Similarly, lactation and feeding services were widely used, particularly in the early postpartum period [65,69,74,78], reflecting both institutional priorities and the central role of milk expression in neonatal care [69,74,78].

In contrast, psychosocial services and postpartum follow-up care were accessed less consistently [71,73,76,77]. Attendance at postpartum follow-up visits was limited across studies [71,77], and a substantial proportion of mothers either did not attend scheduled visits or delayed follow-up beyond the recommended timeframe [71]. Mothers described physical exhaustion [69,77], emotional strain [72,77], competing caregiving responsibilities [73], and logistical barriers such as transportation, cost, and scheduling difficulties [66,71] as factors constraining engagement with ongoing care. In some cases, prioritization of the infant’s medical needs over maternal health further reduced service utilization [69,78].

Perceived adequacy and satisfaction with care were closely linked to relational and communicative aspects of service delivery rather than to service availability alone [67,71,75,76,79]. Care was described as adequate when it was experienced as empathetic, individualized, and characterized by clear, respectful communication [67,71,76]. Feeling listened to and acknowledged as a person, rather than solely as the mother of a patient, emerged as central to positive evaluations of care [75,76]. Conversely, lower perceived adequacy was reported in contexts where communication was inconsistent, guidance contradictory, or emotional needs were perceived as secondary to clinical priorities [67,69,75,79]. Differences in perceived adequacy were reported both within and across healthcare settings [67,75,79], indicating that utilization alone did not ensure that care needs were met.

#### 3.4.4. Contextual and Structural Factors Influencing Care

A range of contextual and structural factors shaped mothers’ experiences of care, patterns of service utilization, and perceived adequacy [66,67,69,71,72,73,75,79]. Structural constraints included financial burden, transportation challenges, insurance limitations, and the logistical demands associated with frequent visits to the NICU [66,71,73,79]. These factors affected not only access to services but also the sustainability of maternal engagement over time [66,71,73].

Contextual factors encompassed NICU level, visiting policies, geographic distance, and culturally prescribed postpartum practices [65,70,73,80]. In some settings, visiting restrictions or culturally specific postpartum customs limited mothers’ physical presence in the NICU, which influenced opportunities for hands-on caregiving, bonding, and interaction with healthcare professionals [65,70,73]. Differences in NICU acuity and hospital resources further contributed to variability in the intensity and type of support available [67,80].

Together, structural and contextual influences interacted with individual maternal needs and service organization, shaping the degree to which mothers could access care, participate in infant care, and experience their postpartum needs as adequately addressed [66,69,73,79].

#### 3.4.5. Unmet Needs and Gaps in Postpartum Care

Despite the range of services described, recurrent gaps in postpartum care were reported across included studies. Information-related gaps were frequently noted and characterized by unclear, delayed, or inconsistent communication from healthcare providers [66,71,75,79]. Gaps in psychosocial support were also highlighted, particularly regarding limited integration of mental health services into routine postpartum and neonatal care [69,73,75,80]. Structural gaps, including financial burden [66], transportation difficulties [66,71], and restricted access to follow-up services [71,73], further contributed to unmet needs.

Notably, unmet needs were observed not only in resource-constrained settings but also in contexts where services were formally available [69,75,80]. This suggests that discrepancies between mothers’ identified needs and the care they received were influenced by service organization, delivery practices, and alignment with maternal experiences rather than service availability alone.

## 4. Discussion

### 4.1. Overview

This systematic review synthesized evidence from 16 studies on postpartum needs of mothers of preterm infants, the utilization and perceived adequacy of available services, and barriers to care. The included studies consistently reported that mothers experienced multidimensional postpartum needs, including challenges in physical recovery, psychological well-being, informational support, maternal role development, and coping or self-care strategies.

Mothers frequently reported high levels of psychological distress, uncertainty, and exhaustion, as well as unmet needs for emotional support and continuous information. Physical recovery constraints were described in several studies as affecting mothers’ ability to be present in the NICU, engage in caregiving activities, and utilize available postpartum services. Although a range of health and support services was formally available, several studies highlighted limitations in accessibility and perceived effectiveness due to structural, informational, psychosocial, or cultural factors.

Maternal satisfaction with care was reported to be influenced primarily by relational aspects, including communication quality, trust, and involvement in infant care, rather than by service availability alone.

### 4.2. Interpretations Findings in the Context of Existing Literature

The included studies demonstrate that mothers of preterm infants face complex and multifaceted postpartum needs, including physical recovery, psychological well-being, informational support, social support, maternal role development, and coping strategies [69,70,71,72,73,74,75,76,77,78,79,80].

Compared with mothers of term infants or those experiencing short neonatal hospitalizations, mothers of preterm infants experience a more prolonged and intensified disruption of the postpartum period [65,66,69,70,71,73,76,80]. Extended NICU stays, ongoing uncertainty regarding infant outcomes, and sustained separation from the infant contribute to cumulative psychological burden and delayed maternal role acquisition [65,66,69,70,71,73,76,80,81,82]. This aligns with evidence showing that psychological distress following preterm birth may persist well beyond the early postpartum phase and is strongly associated with the NICU environment itself [26,69,73,76,80,83].

Despite these heightened needs, the review reveals a clear mismatch between maternal needs and experienced care. While neonatal services are typically highly specialized and well resourced, maternal postpartum care during NICU hospitalization is frequently fragmented, inconsistent, or perceived as secondary [65,67,68,71,73,76,78,79,84]. This mismatch is particularly evident in the limited coordination between neonatal care and maternal-focused postpartum services. Similar patterns have been described in broader NICU research, where maternal health needs are often overshadowed by infant-centered care priorities [69,70,74,75,85].

Overall, these findings indicate that while neonatal care is intensive and well-organized, maternal needs—particularly for psychological support, information, and guidance in caregiving—are inconsistently addressed. This underscores the importance of integrated postpartum services that simultaneously meet maternal and infant needs [26,65,66,69,71,73,76,78,84,85].

### 4.3. The Role of Midwives and System-Level Blind Spots in Postpartum Care

None of the 16 included studies [65,66,67,68,69,70,71,72,73,74,75,76,77,78,79,80] explicitly examined the role of midwives as care providers in NICU-related postpartum care. This absence represents a clear gap in the empirical evidence regarding midwifery involvement in supporting maternal needs during neonatal hospitalization.

To better understand the observed gaps in service utilization and unmet maternal needs, Andersen’s Behavioral Model of Health Service Use can be applied. According to this model, healthcare utilization is influenced by predisposing factors, enabling resources, and perceived or evaluated needs [43]. Across the included studies, mothers consistently reported high needs for psychological support, guidance in caregiving, and continuous information [65,66,69,71,73,76,77]. However, enabling resources, such as structured access to services, continuity of providers, clear communication, and guidance in infant care, were frequently limited or inconsistent [67,68,79]. This pattern helps explain why, despite substantial need, mothers often underutilized postpartum services or perceived them as insufficient.

Similarly, Donabedian’s structure–process–outcome framework provides a lens for interpreting how these care gaps affect maternal outcomes [86]. Structurally, the included studies indicate that postpartum care for mothers of preterm infants is weakly integrated into NICU systems, with professional roles, particularly those of midwives, remaining unclear or absent [65,71,73,78]. At the process level, communication was often inconsistent, psychosocial support limited, and guidance for caregiving insufficient, which contributed directly to mothers’ unmet needs [69,70,76,79]. The outcomes of these structural and process deficiencies included sustained maternal psychological distress, challenges in maternal role development, and lower satisfaction with care, as consistently documented across the studies [66,72,77,80].

Applying these frameworks in conjunction with the empirical findings allows for a systematic explanation of observed gaps in postpartum care. High maternal needs remain unmet because enabling resources are insufficient, and structural and process deficiencies within NICU systems translate directly into adverse maternal outcomes. The absence of midwives in the included studies highlights a structural and conceptual blind spot [86]: despite clear maternal needs, both the organization of services and the continuity of care are insufficient, resulting in underutilization and unmet needs. This interpretation is firmly grounded in the data from the 16 studies and avoids speculative claims, demonstrating how theoretical models can illuminate observed patterns in service delivery without overriding the empirical evidence.

### 4.4. Implications for Practice and Health Policy

The findings highlight the need to explore and reconceptualize postpartum care for mothers of preterm infants as an integrated component of neonatal intensive care. While the included studies did not examine the role of midwives, their recognized expertise in physical recovery, breastfeeding support, emotional adjustment, and maternal role development suggests that exploring their integration into NICU care pathways could be relevant for addressing unmet maternal needs.

From a policy perspective, postpartum care is often framed as a service beginning only after infant discharge. Models that facilitate earlier or continuous involvement of midwives, through NICU-affiliated midwives, shared care approaches, or telehealth support, could potentially enhance coordination, continuity, and utilization of postpartum services.

Cultural sensitivity and contextual adaptation are important considerations. Care models may need to account for social norms, language barriers, and differing expectations regarding motherhood and help-seeking. Early identification of psychological distress and structured referral pathways to psychosocial support could further support maternal well-being.

Overall, this review indicates that mothers of preterm infants experience substantial postpartum needs that are insufficiently addressed within current NICU-centered care systems. The absence of evidence regarding midwifery involvement highlights a structural and conceptual gap. Investigating the potential role of midwives in NICU pathways could contribute to developing more comprehensive and mother-centered postpartum care models.

### 4.5. Implications for Future Research

Future research could more systematically examine the postpartum health needs of mothers of preterm infants during NICU hospitalization. While existing studies provide information on psychological distress, anxiety, and depressive symptoms, few link these outcomes to mothers’ perceived needs, help-seeking behavior, or use of postpartum care services.

Theory-informed quantitative and mixed-methods approaches could help integrate standardized symptom measures with assessments of unmet needs, service accessibility, and perceived adequacy of care. Such studies may provide a more comprehensive understanding of how psychological and physical symptoms translate into concrete support requirements within NICU contexts.

Intervention studies could explore models of integrated, maternal-centered postpartum care, including midwifery-led and psychosocial support approaches. Outcomes could include not only symptom reduction but also continuity of care, maternal role development, service utilization, and satisfaction with care.

Further research could also address structural and organizational determinants of care, such as coordination between obstetric, neonatal, and postnatal services and professional role integration. Longitudinal studies following mothers from early postpartum through post-discharge could capture the evolving relationships between symptom trajectories, unmet needs, and service utilization over time.

### 4.6. Strengths and Limitations

This review has several strengths. The search strategy was comprehensive and systematic, allowing the inclusion of both qualitative and quantitative studies. Transparent quality and certainty assessments were conducted, enabling a nuanced synthesis of postpartum needs and gaps in care for mothers of preterm infants.

Several limitations should be acknowledged. The availability and heterogeneity of quantitative studies limit the generalizability of some findings. Only peer-reviewed literature was included, and a substantial number of potentially relevant studies could not be accessed in full text, which may have introduced selection bias. Study selection and data extraction were conducted by a single reviewer, which may have further contributed to bias. Finally, the diversity of healthcare settings, cultural contexts, and study designs introduces heterogeneity that may limit direct comparability across studies.

## 5. Conclusions

Mothers of preterm infants experience complex and multidimensional postpartum health needs that are frequently insufficiently addressed within existing care structures. This review highlights the importance of considering integrated, accessible, and psychosocially informed postpartum care models. Recognizing maternal health alongside infant care may support maternal well-being and potentially contribute to family functioning and long-term outcomes for preterm infants.

## Figures and Tables

**Table 1:** Inclusion and Exclusion Criteria.

Category	Criterion	Inclusion	Exclusion
S	Target population	Mothers of preterm infants (<37 weeks gestation)	Fathers, other family members, mixed parent groups without separate analysis of mothers; healthcare professionals
	Setting	Hospitalization in NICU/Neonatology/Perinatal center	Post-discharge period
	Region	Worldwide	No exclusion
	Age	All age groups	No exclusion
PI	Health needs	Physical, psychological, emotional, informational needs.	Child-only needs
	Health-related services use	Use of health services, psychosocial services, counseling, care, perceived support for needs	Use of exclusively pediatric/neonatal services
	Experiences	Perception	
D	Design	- Qualitative studies (interviews, focus groups, ethnography) - Quantitative studies (needs assessments, surveys, questionnaires) - Observational studies - Mixed-methods	- Editorials, commentaries, opinion pieces - Purely biomedical child outcomes - Intervention studies - Systematic reviews (only for manual search)
E	Outcomes	- (Unmet) needs - Usage patterns - Barriers - Perceived support gaps - Emotional/psychological burdens related to care	- Child outcomes without maternal perspective - Purely objective registry data without subjective assessments - Only prevalence/statistics on diseases or sociodemographic characteristics
R	Research type	- Qualitative, quantitative, mixed-methods primary studies - Peer-reviewed publications	- Reviews, protocols, commentary literature - Conference abstracts, dissertations/theses, grey literature

**Table 2:** Search strategy and results for each database.

Database	Syntax	Number
PubMed/ MEDLINE	((“Mothers”[Mesh] OR mother* OR maternal* OR “maternal health” OR “maternal experience”) AND (“Infant, Premature”[Mesh] OR “preterm infant*” OR “premature infant*” OR “preterm neonate*” OR “premature neonate*” OR “preterm baby*” OR “premature baby*”) AND (“Intensive Care, Neonatal”[Mesh] OR NICU OR “neonatal intensive care unit” OR “neonatal ward”) AND (health need* OR need* OR support* OR “psychological need*” OR coping* OR “health behavior” OR self-care OR utilization OR “service use” OR “healthcare access”) AND (postpartum OR postnatal))	482
CINHAL	((MH “Mothers” OR MH “Maternal Health” OR mother* OR maternal* OR “maternal experience”) AND (MH “Infant, Premature” OR “preterm infant*” OR “premature infant*” OR “preterm neonate*” OR “premature neonate*” OR “preterm baby*” OR “premature baby*”) AND (MH “Neonatal Intensive Care Units” OR NICU OR “neonatal intensive care unit” OR “neonatal ward”) AND (health need* OR need* OR support* OR “psychological need*” OR coping* OR “health behavior” OR self-care OR utilization OR “service use” OR “healthcare access”) AND (postpartum OR postnatal))	137
APA PsycINFO	((DE “Mothers” OR DE “Maternal Role” OR mother* OR maternal* OR “maternal experience”) AND (DE “Preterm Infant” OR “preterm infant*” OR “premature infant*” OR “preterm neonate*” OR “premature neonate*” OR “preterm baby*” OR “premature baby*”) AND (DE “Neonatal Intensive Care Units” OR NICU OR “neonatal intensive care unit” OR “neonatal ward”) AND (health need* OR need* OR support* OR “psychological need*” OR coping* OR “health behavior*” OR self-care OR utilization OR “service use” OR “healthcare access”) AND (postpartum OR postnatal))	81
Web of Science	TS = (mother* OR maternal* OR “maternal health” OR “maternal experience”) AND TS = (“preterm infant*” OR “premature infant*” OR “preterm neonate*” OR “premature neonate*” OR “preterm baby*” OR “premature baby*”) AND TS = (NICU OR “neonatal intensive care unit” OR “neonatal ward”) AND TS = (“health need*” OR need* OR support* OR “psychological need*” OR coping* OR “health behavior*” OR self-care OR “service use*” OR “healthcare access”) AND TS = (postpartum OR postnatal)	313

**Table 3:** Methodological quality and risk of bias of included studies.

Study	Checklist	Criteria Met	Quality
Abukari et al. (2025) [65]	CASP Qualitative Checklist	10/10	High
Adu-Bonsaffoh et al. (2022) [66]	CASP Qualitative Checklist	10/10	High
Alghamdi et al. (2025) [67]	CASP Qualitative Checklist	10/10	High
Amankwaa, Pickler & Boonmee (2007) [68]	CASP Cohort Study Checklist	8/12	Moderate
Brockway et al. (2020) [69]	CASP Qualitative Checklist	9/10	High
Chang Lee et al. (2009) [70]	CASP Qualitative Checklist	9/10	High
Chen et al. (2025) [71]	CASP Qualitative Checklist	9/10	High
Daliri et al. (2024) [72]	CASP Qualitative Checklist	9/10	High
Fowler et al. (2019) [73]	CASP Qualitative Checklist	8/10	Moderate
Gianni et al. (2018) [74]	Cross-Sectional Studies Checklist	7/11	Moderate
Goutaudier et al. (2011) [75]	MMAT	2/5	Low
	CASP Qualitative Checklist	9/10	High
	Cohort Study Checklist	6/10	Moderate
Herzberg et al. (2016) [76]	CASP Qualitative Checklist	9/10	High
Jiyun & Kyung-Sook (2022) [77]	Cohort Study Checklist	10/11	High
Palmquist et al. (2020) [78]	CASP Qualitative Checklist	8/10	Moderate
Witt et al. (2022) [79]	CASP Qualitative Checklist	9/10	High
Woodward et al. (2014) [80]	Cohort Study Checklist	10/12	High

## Data Availability

No new data were created or analyzed in this study. Data sharing is not applicable to this article.

## Abbreviations

The following abbreviations are used in this manuscript:

NICU	Neonatal intensive care unit
GA	Gestational age
LBW	Low birth weight
VLBW	Very low birth weight
PTSD	Post-traumatic stress disorder
IUGR	Intrauterine Growth Restriction
AWMF	Association of the Scientific Medical Societies in Germany
WHO	World Health Organization

## Appendix A

**Table A1:** Description of the included studies.

Author/Year	Country/Region	Setting	NICU-Level	Sample	Gestational Age/Birth Weight	Study Objective	Design/Method	Outcomes
Abukari et al. [65]	Ghana/Accra	Korle-Bu Teaching Hospital/during NICU hospitalization	Level III	*n* = 25; Education: no formal–college; Residence: KBTH catchment	<37 GA	Experiences, support needs, coping strategies	Qualitative; focus groups; semi-structured; thematic content analysis	Psychosocial support, coping, barriers
Adu-Bonsaffoh et al. [66]	Ghana/Accra	Korle-Bu Teaching Hospital/early postnatal period	Level III	*n* = 50; Age 20–30 (50%); JHS 43.3%; married/cohabiting 63.3%; petty traders 50%	<37 GA	Knowledge and experiences of premature birth	Qualitative; phenomenological; in-depth interviews; thematic analysis	Experiences, support, needs
Alghamdi et al. [67]	Saudi Arabia/Eastern Province	Online (Zoom)/infant <1 year	N/A	*n* = 5; Age 26–37; Education HS–PhD; 7 infants; 2 twins	<37 GA	Realities of life, emotional challenges	Qualitative; phenomenological; in-depth interviews; hybrid thematic analysis	Emotional challenges, support systems
Amankwaa, Pickler & Boonmee [68]	USA	University Medical Center/6 w pp., 2 w post-discharge, 3 m pp.	Level III	*n* = 23; Age 18–42; mostly low income; diverse education	<37 GA	Stress, support, self-esteem	Quantitative; longitudinal; EPDS, RS, Well-Being Scale, MAQ, MIRI	Stress, well-being, support
Brockway et al. [69]	Canada/Alberta	Five Level II NICUs	Level II	*n* = 14; mean age 29.7; 64% primiparous; high income	32 + 0–34 + 6 GA; >1500 g	Experiences with infant nutrition	Qualitative; semi-structured interviews; thematic analysis	Experience, support, breastfeeding
Chang Lee et al. [70]	Taiwan	Major neonatal care centre/pre-discharge	N/A	*n* = 26; GA 25–34 w; BW 530–1490 g; prolonged NICU stay	VLBW < 1500 g	Parental experiences, bonding	Qualitative; grounded theory; interviews + observation	Emotional challenges, support
Chen et al. [71]	USA/California	University Hospital/NICU or phone/14–55 d pp.	Level IV	*n* = 26; median age 28–32; mixed parity; mostly public insurance	<37 GA	Postnatal care needs	Qualitative; semi-structured interviews; thematic analysis	Needs, perception, support
Daliri et al. [72]	Ghana/Upper East	Regional Hospital NICU ≥48 h	N/A	*n* = 13; majority 20–29 y; low education; varied occupations	<37 GA	Psychosocial experiences	Qualitative; semi-structured interviews; Colaizzi analysis	Emotional responses, coping
Fowler et al. [73]	Australia	Telephone interviews/<6 m post-discharge	N/A	*n* = 10; GA 24–27 w; BW 490–1160 g; long NICU stay	24–27 GA	NICU and transition home experiences	Qualitative; semi-structured interviews; thematic analysis	Experiences, coping, support
Gianni et al. [74]	Italy/Milan	Tertiary hospital/24 h pre-discharge	Level III	*n* = 64; mean GA 29.9 w; BW 1357 g; 25% twins	≤33 GA	Breastfeeding barriers/facilitators	Quantitative; cross-sectional; questionnaire	Support use, barriers
Goutaudier et al.—qualitative component [75]	France/South	Ward or accommodation/during NICU stay	N/A	*n* = 27; GA 27–37 w; NICU stay 2–9 w	27–37 GA	Psychological consequences	Mixed-methods; interviews + EPDS	Stress, psychological strain
Goutaudier et al.—quantitative component [75]	France/South	Ward or accommodation/during NICU stay	N/A	*n* = 27; GA 27–37 w; NICU stay 2–9 w	27–37 GA	Psychological consequences	Mixed-methods; interviews + EPDS	Stress, psychological strain
Herzberg et al. [76]	Germany/Berlin	Charité/post-discharge	Level III–IV	*n* = 5; mean age 36; twins common; NICU ≥ 4 w	ELBW < 1000 g	Subjective support needs	Qualitative; interviews; metaphor & content analysis	Needs, coping, support
Park & Bang [77]	South Korea	Tertiary hospital	Level III	*n* = 91; mean age 35; primiparous common; NICU visits daily; multiple births; Infants; Mean gestational age: 31.8 weeks; mean birth weight: 1489.5 g	< 37 GA	Physical and emotional health status	Quantitative; questionnaire (self-reported questionnaire; EPDS; STAI; Guilt)	Emotional and physical needs
Palmquist et al. [78]	USA	Single NICU + online/in-person	N/A	*n* = 17; age 20–39; primiparous & multiparous; NICU across USA	VLBW < 1500 g	Experiences with breastfeeding/breast pumping	Qualitative; interviews; thematic analysis	Experiences, coping, support
Witt et al. [79]	USA/Massachusetts, Missouri, and Colorado	Five Hospitals with Level III-IV	Level III-IV	*n* = 39; Age 18–42; mostly low income; diverse education	<37 GA	Stress, support, self-esteem	Quantitative; longitudinal; EPDS, RS, Well-Being Scale, MAQ, MIRI	Stress, well-being, support
Woodward et al. [80]	Canada/Alberta	Christchurch Women’s Hospital	Level III	*n* = 133; mean age 29.7; 64% primiparous; high income	32 + 0–34 + 6 GA; >1500 g	Experiences with infant nutrition	Qualitative; semi-structured interviews; thematic analysis	Experience, support, breastfeeding

## Appendix B

**Table A2:** Summary of certainty of evidence assessment.

Study	Design	Evidence Framework	Evidence Unit Assessed	Methodological Limitations	Coherence/Consistency	Adequacy/Precision	Relevance/Indirectness	Publications Bias	Overall Certainty
Abukari et al. [65]	Qualitative (FGDs)	GRADE-CERQual	Review findings (burden, support, coping)	Minor	High	Adequate	High	—	Moderate–High
Adu-Bonsaffoh et al. [66]	Qualitative (IDIs)	GRADE-CERQual	Review findings (experiences, care)	Moderate	High	Moderate	Moderate–High	—	Moderate
Alghamdi et al. [67]	Qualitative	GRADE-CERQual	Review findings (experience, coping)	Moderate	High	Moderate	High (context-specific)	—	Moderate
Amankwaa, Pickler & Boonmee [68]	Observational (longitudinal)	GRADE	Maternal responsiveness	Serious	Consistent	Imprecise	Direct	Unclear	Low–Moderate
Brockway et al. [69]	Qualitative	GRADE-CERQual	Review findings (breastfeeding self-efficacy)	Minor	High	Adequate	High	—	Moderate
Chang Lee et al. [70]	Qualitative (Grounded theory)	GRADE-CERQual	Theoretical model	Minor–Moderate	High	Moderate–High	Moderate	—	Moderate–High
Chen et al. [71]	Qualitative	GRADE-CERQual	Review findings (postpartum care)	Minor–Moderate	High	Adequate	High	—	Moderate
Daliri et al. [72]	Qualitative	GRADE-CERQual	Review findings (psychosocial experiences)	Minor–Moderate	High	Adequate	High (LMIC context)	—	Moderate–High
Fowler et al. [73]	Qualitative	GRADE-CERQual	Review findings (maternal burden)	Minor–Moderate	High	Moderate	High	—	Moderate
Gianni et al. [74]	Observational (cross-sectional)	GRADE	Breastfeeding facilitators/barriers	Serious	N/A	Moderate	Direct	Unlikely	Low–Moderate
Goutaudier et al.—qualitative component [75]	Qualitative	GRADE-CERQual	Review findings (trauma, support, peer coping)	Moderate	High	Adequate	High	—	Moderate
Goutaudier et al.—quantitative component [75]	Observational (cross-sectional)	GRADE	EPDS ≥ 12; PTSD symptoms (IES-R)	Serious	N/A	Imprecise	Indirect	Likely	Low
Herzberg et al. [76]	Qualitative	GRADE-CERQual	Review findings (support needs)	Moderate	High	Moderate	High	—	Moderate
Park & Bang [77]	Qualitative	GRADE-CERQual	Experiences and coping (review findings)	Moderate	High	Moderate	High (context-specific)	—	Moderate
Palmquist et al. [78]	Qualitative (ethnography)	GRADE-Equal	Review findings (embodiment, lactation)	Minor–Moderate	High	Moderate	High	—	Moderate
Witt et al. [79]	Qualitative (secondary analysis)	GRADE-CERQual	Review findings (stress)	Minor	High	Adequate	High	—	High
Woodward et al. [80]	Observational (prospective)	GRADE	Maternal stress & development	Moderate	Consistent	Moderate	Some indirectness	Unlikely	Moderate

## Appendix C

**Table A3:** Matrix of maternal experiences and needs following preterm birth.

Study	PH	PSY	INFO	SOC	COP	USE	BAR
Abukari et al. [65]	—	“I felt a deep sense of fear and helplessness.” (FG2M1)	“They, … provided me with the information I needed to understand my baby’s condition.” (FG5M2)	“My parents helped financially and emotionally.” (FG4M2)	“Learning how to use the feeding tubes and pumps was intimidating at first, but, … I became confident.” (FG1M4)	“Talking to the nurses and doctors helped a lot.” (FG5M2)	“The limited access to my baby was difficult, … the hospital had strict visiting hours (FG5M3)
Adu-Bonsaffoh et al. [66]	“If you bend down too much, over stressed, … plenty things like that can cause it.” (Married, 30 years)	“I feel very sad, … there is no mother who would feel happy when she is somewhere else and her baby [is] at another end.” (Married, 28 years)	“If they can carry out research into it… and educate us, I’m sure these things (preterm deliveries) wouldn’t go on.” (Cohabiting, 27 years)	“All I saw was ward fee and oxygen. When I visited the baby on the second day I didn’t see any oxygen being given to him so I asked her why I was charged for oxygen and she said the baby was given. She brought the confirmation which had been recorded that the baby was on oxygen for 24 h.” (Married, 38 years)	“I was scared when they took the baby there [NICU] because my first pregnancy. That pregnancy was [ended at] about 26 weeks. They took the baby there and the next day the baby passed on. So this one I was so scared. I was praying to God that, ‘please this one spare me’.” (Single, 27 years)	“I live far from here [the hospital] and I come here and see the baby three times a day.” (Married, 28 years)	“I have had CS and having to walk up and down is very worrying for me.” (Married, 38 years)
Alghamdi et al. [67]	—	“I was very afraid for my child.” (Participant 4)	“I needed full information about my baby, … I did not want to feel like I did not know what was going on.” (Participant 1)	“The factors that helped me the most were my family and my husband and his support.” (Participant 4)	“God will plan goodness for me, and everything will be settled by God.” (Participant 1)	“The medical care was excellent. They calmed me down a lot, … Even the nurses were nice.” (Participant 1)	“Individuals, for instance, whose behavior or style lacks manners … Her behavior was terrible. It mattered to me how she handled things.” (Participant 2)
Amankwaa, Pickler & Boonmee [68]	—	“… scores for depression, social support, stressors, self-esteem, maternal well-being, attitude, and responsiveness did not differ significantly from one time to the next, … ”	—	“… Mothers in this sample had relatively high levels of well-being, social support, self-esteem, and maternal responsiveness …”	—	—	“… mothers reported relatively high levels of everyday stressors, they did not report high levels of depression, …”
Brockway et al. [69]	“I just looked like death because I wasn’t sleeping, … That went on for 3 months of me just getting hour and a half bursts of sleep.” (15—negative changes in BSES)	“That breastfeeding agenda really, really just kind of ruined … the first few months with my daughter, … I missed the newborn stage.” (15—negative changes in BSES)	“I met with the lactation consultant and she gave me some tips about increasing my milk supply.” (06—positive changes BSES)	“The overarching concept from the NICU environment was the primacy of the infant over the mental and physical health needs of the mother.” (5.1 NICU environment)	“Honestly, the most support I got aside from my husband would have been the nurses in the NICU. They were absolutely phenomenal …” (10	“Honestly, the most support I got aside from my husband would have been the nurses in the NICU. They were absolutely phenomenal, …” (10)	“”Some mothers were unable to be physically present at the bedside for feeding times due to their own health conditions or social situations.” (5.1.1 NICU environment)
Chang Lee et al. [70]	“I know that coming every day for the kangaroo care for my baby would be bad for my health, … because I didn’t practice my Zuo Yue Zi fully, but, … I’d rather be with my baby and hold her in my arms.” (M7)	“In the beginning, I felt that it wasn’t really happening, … I never thought I would have a tiny baby who was nothing like the baby I normally saw and envisaged for me.” (M5)	“No one told me what I could do for my baby, … they should at least have told me what to do and given me some instructions.” (M2)	“Being a mother is ‘knowing your child’.” (M13)	“I made tapes when I missed him. I wanted him to get to know my voice and me. it made me feel that I was with him.” (M17)	“They [the nurses] are friendly and they talked me through holding my baby for the first time. it made the whole thing much easier.” (M20)	“When I walked into this big room with all the incubators and all the other critically ill little babies, I couldn’t focus on just mine. There were so many machines sending out loud beeps. I just wanted to run away.” [M2]
Chen et al. [71]	“Participants all had medical concerns related to their pregnancy, delivery, or postpartum recovery process to discuss with their physician, such as assessment of physical recovery after birth, counseling about nutrition after childbirth, assessment of postpartum mood, review of pregnancy events, future pregnancy planning, and breastmilk feeding and infant care.”	“Because of all my emotions and not knowing what is what, I do feel like it’s very important (to see the doctor after delivery).” (Participant 3)	“It wasn’t really more of, like, a sit down, let’s discuss, … mostly I think we just discussed how my incision was looking.” (Participant 5)	“For those who attended a postpartum visit, certain aspects of their visit produced a more positive experience, such as when the clinician created a safe space for asking questions and did not make the participants feel rushed for time.”	“Instead of relying on the survey tool to identify postpartum depression, participants reflected on ways they were differentiating between an overwhelming experience of having a preterm infant in the NICU and postpartum depression for themselves.”	“Eight (31%) participants had attended a postpartum visit; four (15%) were not planning to attend a postpartum visit … The remaining 14 (54%) had an upcoming appointment scheduled.”	“Eight (31%) participants had attended a postpartum visit; four (15%) were not planning to attend a postpartum visit, … The remaining 14 (54%) had an upcoming appointment scheduled.”
Daliri et al. [72]	—	“After the CS, I was told that my baby would be born premature and I was scared, … this put so much fear in me.” (P006, 5 days on admission)	—	“Sometimes I depend on my relatives for support, … They have been very helpful.” (P001, 7 days on admission)	“Now I don’t feel sad because I have given everything to God, … so I am just praying to God and trusting that my baby will be fine and we will be discharged.” (P013, 14 days on admission)	“Some of the medical team members especially the nurses supported me. They gave me some diapers for my preterm baby.” (P002, 4 days on admission)	“There is not enough space in the NICU, and there are not enough beds for admissions, so I didn’t get a bed to sleep so I had to sleep on the floor.” (P005, 3 days on admission)
Fowler et al. [73]	“I was in pain for the first few days and couldn’t stand up to do any of his care.” (M8)	“It’s really stressful because all you want is for your child to be okay, … and no one is going to give you the answers for that.” (M3)	“No one told us what it meant to have an extremely premature baby.” (M8)	“They [postnatal ward mothers] got a lot more support than we did.” (M8)	“The extreme difference from the hospital setting to home setting can, … It really, I spiraled into massive anxieties, which I’m only just getting under control now, through seeing a psychologist, …”(M6)	“No, … no I’m fine now I did talk to the home nurse who came to visit she offered a group, … I had a really high powered job and then I was stuck home with [baby], … As I said I’m fine now, … I did have some things I had to work through, … So I’m fine.” (M8)	“Being in [hospital] was a very isolating experience, … then to come home and be so far from everyone, emotionally that was quite hard.” (M4)
Gianni et al. [74]	—	Stress/anxiety as barrier (18%)	Info on infant suckling evaluation only provided to 50%	Lactation consultant support within first 8 h (38%)	—	“Although most of the mothers felt that the health care professionals in the NICU were supportive with regard to breastfeeding, …”	Pumping initiated within 24 h (58%)
Goutaudier et al. [75]	“I need to go home at night.”	“It’s my fault, … I felt like the worst mother in the world!”	“There was nothing about the caesarean, it’s a pity.”	“My husband? He is like Superman, …”	“Twelve women looked for information in order to be able to cope with any eventuality: ‘It’s my own way to fight’.”	“Fifteen women went to discussion groups, … ‘therapeutic’.”	“Five women reported a negative representation of the medical staff and considered that they received no support: ‘What is wrong with these people? Do they have hearts under these white coats?’”
Herzberg et al. [76]	“The mothers described their situation as a prolonged state of inner tension characterized by physical and psychological exhaustion.” [translated from German]	“The fear of losing orientation and control in the unfamiliar intensive care environment becomes a constant companion.” [translated from German]	“Gradually, they want to know more about the medical status and health condition of their child, about routine ward procedures and planned interventions.” [translated from German]	“Through guidance and training, they want to learn how to handle their preterm infant in order to actively fulfil their maternal role.” [translated from German]	“There, in parental counselling, you could release your emotions, … I felt strengthened and safe afterwards.” [translated from German]	“Positive was how we, as parents, were involved from the beginning, step by step, in the care of our child.” [translated from German]	“I felt that most of the nurses thought I was stupid because I wanted to breastfeed my child exclusively.” [translated from German]
Park & Bang [77]	Fatigue M = 3.42; shoulder pain M = 3.12 (symptom scale 1–5)	EPDS M = 11.02; 44% at risk (EPDS 0–30)	80.2% did not receive postpartum health education	“Grandparents were the most common additional caretakers, with 39 mothers (42.8%) answering that grandparents helped care for the baby”	—	Postpartum caretaker 28 (30.8%) (Assistance from other caretakers)	80.2% did not receive postpartum health education
Palmquist et al. [78]	“I know you’re not feeling well, but if you want milk, you have to pump.”	“It was probably the worst day of my life, … extremely traumatic, …”	“It wasn’t really discussed with us what the benefits or risks were, …”	“Not having the opportunity to do things for your own child makes you feel less like a parent.”	“Everyone worked together and included us in decisions.”	“Supportive lactation consultants… helped me resist the doctors’ decisions.”	“Neonatologists generally were portrayed as ‘ambivalent about breastfeeding” at best, or actively discouraging at it at worst”
Witt et al. [79]	“So many painkillers, … after the two surgeries my wound got infected…”	“My baby is struggling to breathe, … I was just screaming.”	“Nobody ever talked to me in Spanish here, …”	“I didn’t feel that, … until maybe a month, maybe two months into the NICU, …”	“It’s just him and me and my children to support each other.”	“I didn’t really get in touch with the doctors until the end. You only see the nurses “	“I used the translator on the phone, …”
Woodward et al. [80]	—	Depressive symptoms increased NICU stress (*p* < 0.001); low parenting confidence increased stress (*p* = 0.002)	—	—	—	“Least stressful scores were issues related to staff behavior and communication (M = 1.83)”	Separation (M = 4.04); helplessness protecting infant (M = 3.83); helplessness helping infant (M = 3.41); PSS: NICU 1–5

PH = Physical health and physical recovery; PSY = Psychological and emotional experiences; INFO = Information needs and communication; SOC = Social support (family, peers, professionals); COP = Coping strategies; USE = Use of healthcare services and support resources; BAR = Barriers.

## References

[1] Pavlyshyn H., Sarapuk I., Saturska U. (2024). Maternal Stress Experience in the Neonatal Intensive Care Unit after Preterm Delivery. Am. J. Perinatol..

[2] Holditch-Davis D., Santos H., Levy J., White-Traut R., O’Shea T.M., Geraldo V., David R. (2015). Patterns of psychological distress in mothers of preterm infants. Infant Behav. Dev..

[3] Verbiest S., Ferrari R., Tucker C., McClain E.K., Charles N., Stuebe A.M. (2020). Health Needs of Infants in a Neonatal Care Unit: A Mixed-Methods Study. Ann. Intern. Med..

[4] Euro-Peristat Network (2022). European Perinatal Health Report: Core Indicators of the Health and Care of Pregnant Women and Babies in Europe 2015–2019.

[5] Institut für Qualitätssicherung und Transparenz im Gesundheitswesen (IQTIG) Bundesauswertung PM-GEBH: Geburtshilfe—Auswertungsjahr 2024. https://iqtig.org/downloads/auswertung/aj2024/pm-gebh/DeQS-RL_PM-GEBH_AJ2024_BUAW_V02_2024-08-15.pdf.

[6] Berger R.H., Hösli I., Schneider S., Surbek D. (2022). Die Frühgeburt: Prädiktion, Prävention und Management. eMedpedia—Die Geburtshilfe.

[7] Bramer G.R. (1988). International statistical classification of diseases and related health problems. Tenth revision. World Health Stat. Q..

[8] Ndjomo J., Njiengwe P., Moudze B., Guifo O., Blairy S. (2025). Posttraumatic stress, anxiety, and depression in mothers after preterm delivery and the associated psychological processes. BMC Pregnancy Childbirth.

[9] Romero R., Dey S.K., Fisher S.J. (2014). Preterm labor: One syndrome, many causes. Science.

[10] Haas D.M., Caldwell D.M., Kirkpatrick P., McIntosh J.J., Welton N.J. (2012). Tocolytic therapy for preterm delivery: Systematic review and network meta-analysis. BMC.

[11] Satyaraddi A., Sooragonda B.G., Satyaraddi A.A., Khadilkar K., Ks S., Kiran L., Kannan S. (2024). Antenatal Corticosteroids and Their Effects on Maternal Glycemic Status: A Prospective Observational Study from an Indian Tertiary Referral Center. Cureus.

[12] Jellyman J.K., Fletcher A.J., Fowden A.L., Giussani D.A. (2020). Glucocorticoid Maturation of Fetal Cardiovascular Function. Trends Mol. Med..

[13] Abdel-Aleem H., Shaaban O.M., Abdel-Aleem M.A. (2013). Cervical pessary for preventing preterm birth. Cochrane Database Syst. Rev..

[14] Mogos M.F., August E.M., Salinas-Miranda A.A., Sultan D.H., Salihu H.M. (2013). A Systematic Review of Quality of Life Measures in Pregnant and Postpartum Mothers. Appl. Res. Qual. Life.

[15] Thanh B.Y., Lumbiganon P., Pattanittum P., Laopaiboon M., Vogel J.P., Oladapo O.T. (2019). Mode of delivery and pregnancy in preterm birth: A secondary analysis of the WHO Global and Multi-country Survery. Sci. Rep..

[16] Roberts D., Brown J., Medley N., Dalziel S.R. (2017). Antenatal corticosteroids for accelerating fetal lung maturation for women at risk of preterm birth. Cochrane Database Syst. Rev..

[17] Pacagnella R.C., Cecatti J.G., Parpinelli M.A., Sousa M.H., Haddad S.M., Costa M.L. (2014). Delays in receiving obstetric care and poor maternal outcomes: Results from a national multicentre cross-sectional study. BMC Pregnancy Childbirth.

[18] Arbeitsgemeinschaft der Wissenschaftlichen Medizinischen Fachgesellschaften (AWMF) (2022). Prävention und Therapie der Frühgeburt (S2k-Leitlinie 015-025).

[19] Zeitlin J., Maier R.F., Cuttini M., Aden U., Boerch K., Gadzinowski J., Jarreau P.-H., Lebeer J., Norman K., Pedersen P. (2020). EPICE and SHIPS Research Group Cohort Profile: Effective Perinatal Intensive Care in Europe (EPICE) very preterm birth cohort. Int. J. Epidemiol..

[20] Maier R.F., Blondel B., Piedvache A., Misselwitz B., Petrou S., Van Reempts P., Franco F., Barros H., Gadzinowski J., Boerch K. (2018). Duration and Time Trends in Hospital Stay for Very Preterm Infants Differ Across Europe Regions. Pediatr. Crit. Care Med..

[21] Arbeitsgemeinschaft der Wissenschaftlichen Medizinischen Fachgesellschaften (AWMF) (2018). Psychosoziale Betreuung von Familien mit Früh- und Neugeborenen.

[22] O’Brien K., Robson K., Bracht M., Cruz M., Lui K., Alvaro R. (2018). Effectiveness of Family Integrated Care in neonatal intensive care units on infant and parent outcomes: A multicentre, multinational, cluster-randomised controlled trial. Lancet Child Adolesc. Health.

[23] Hohmann M. (2010). Wochenbett—Physiologie und Pathologie. Frauenheilkd. Up2date.

[24] Cunningham F.G., Leveno K.J., Dashe J.S., Hoffman B.L., Spong C.Y., Casey B.M. (2022). The Puerperium. Williams Obstetrics.

[25] Ross L.E., Murray B.J., Steiner M. (2005). Sleep and perinatal mood disorders: A critical review. J. Psychiatry Neurosci..

[26] O’Hara M.W., McCabe J.E. (2013). Postpartum depression: Current status and future directions. Annu. Rev. Clin. Psychol..

[27] Dong D., Ru X., Sang T., Li S., Wang Y., Feng Q. (2022). A prospective cohort study on lactation status and breastfeeding challenges in mothers giving birth to preterm infants. Int. Breastfeed. J..

[28] Reddy U.M., Rice M.M., Grobman W.A., Bailit J.L., Wapner R.J., Varner M.W., Throp J.M., Leveno K.J., Caritis S.N., Prasad M. (2015). Serious maternal complications after early preterm delivery (24–33 weeks’ gestation). Am. J. Obstet. Gynecol..

[29] World Health Organization (2022). WHO Recommendations on Postnatal Care of the Mother and Newborn.

[30] Campbell O.M., Cegolon L., Macleod D., Benova L. (2015). Length of Stay After Childbirth in 92 Countries and Associated Factors in 30 Low- and Middle-Income Countries: Compilation of Reported Data and a Cross-sectional Analysis from Nationally Representative Surveys. PLoS Med..

[31] Hockamp N., Sievers E., Hulk P., Rudolff H., Rudolff S., Lücke T., Kersting M. (2022). The role of breastfeeding promotion in German hospitals for exclusive breastfeeding duration. Matern. Child Nutr..

[32] Deutscher Hebammenverband (2025). Vertrag Über Die Versorgung Mit Hebammenhilfe Nach §134a SGB V..

[33] Hertle D., Wende D., von Sayn-Wittgenstein F. (2024). Aufsuchende Wochenbettbetreuung: Die sozioökonomische Lage hat einen starken Einfluss auf den Betreuungsumfang Eine Analyse mit Routinedaten der BARMER. Gesundheitswesen.

[34] Knobloch-Maculuve K., Steinhäuser J. (2024). Versorgung von Familien im Wochenbett—Eine qualitative Studie. Z. Allg..

[35] Edmaier H., Pehlke-Milde J. (2024). Access to midwifery care—The perspective of women in life situations with psychosocial stress factors. GMS Z. Hebammenwiss..

[36] Stahl K. (2019). Prädiktoren der intra- und postpartalen Betreuungserfahrung: Schlüsseldimensionen einer guten Betreuung aus Sicht der Gebärenden und Wöchnerinnen. GMS Z. *Hebammenwiss*..

[37] von Rahden O. (2019). Betreuung von Frühgeborenen und Neugeborenen mit Erkrankungen von Schwangerschaft bis Wochenbett. Hebamme.

[38] von Rahden O. (2019). Betreuung von Frühgeborenen und Neugeborenen mit Erkrankungen von Schwangerschaft bis Wochenbett. Hebamme.

[39] Lederman R.P., Boyd A., Pitts K., Roberts-Gray C., Hutchinson M., Blackwell S. (2013). Maternal development experiences of women hospitalized to prevent preterm birth. Sex. Reprod. Healthc..

[40] Alizadeh-Dibazari Z., Abdolalipour S., Mirghafourvand M. (2023). The effect of prenatal education on fear of childbirth, pain intensity during labour and childbirth experience: A scoping review using systematic approach and meta-analysis. BMC Pregnancy Childbirth.

[41] Vanderlaan J., Sadler L., Kjerulff K. (2021). Association of Delivery Outcomes with the Number of Childbirth Education Sessions. J. Perinat. Neonatal Nurs..

[42] Mari K.E., Hoke M.K., Darden N., Burris H.H. (2025). Postpartum care receipt among parents of infants admitted to a freestanding children’s hospital neonatal intensive care unit (NICU). J. Perinatol..

[43] Andersen R.M. (1995). Revisiting the behavioral model and access to medical care: Does it matter?. J. Health Soc. Behav..

[44] Page M.J., McKenzie J.E., Bossuyt P.M., Boutron I., Hoffmann T.C., Mulrow C.D., Shamseer L., Tetzlaff J.M., Akl E.A., Brennan S.E. (2021). The PRISMA 2020 statement: An updated guideline for reporting systematic reviews. BMJ.

[45] Popay J., Roberts H., Sowden A., Petticrew M., Arai L., Rodgers M., Britten N., Roen K., Duffy S. (2006). Guidance on the Conduct of Narrative Synthesis in Systematic Reviews: A product from the ESRC Methods Programme.

[46] Critical Appraisal Skills Programme. CASP Checklists. https://casp-uk.net/casp-tools-checklists/.

[47] Hong Q.N., Pluye P., Fàbregues S., Bartlett G., Boardman F., Cargo M., Dagenais P., Gagnon M.-P., Griffiths F., Nicolau B. Mixed Methods Appraisal Tool (MMAT), Version 2018. http://mixedmethodsappraisaltoolpublic.pbworks.com/w/file/fetch/127916259/MMAT_2018_criteria-manual_2018-08-01_ENG.pdf.

[48] Hultcrantz M., Rind D., Akl E.A., Treweek S., Mustafa R.A., Iorio A., Alper B.S., Meerpohl J.J., Murad M.H., Ansari M.T. (2017). The GRADE Working Group clarifies the construct of certainty of evidence. J. Clin. Epidemiol..

[49] Lewin S., Glenton C., Munthe-Kaas H., Carlsen B., Colvin C.J., Gülmezoglu M., Noyes J., Booth A., Garside R., Rashidian A. (2015). Using qualitative evidence in decision making for health and social interventions: An approach to assess confidence in findings from qualitative evidence syntheses (GRADE-CERQual). Implement. Sci..

[50] Karsch J., Schönfeld M., Mühler A.K., Tippmann S., Arnold C., Urschitz M.S., Mildenberger E., Kidszun A. (2025). Trajectory of parental health-related quality of life after neonatal hospitalization—A prospective cohort study. Health Qual. Life Outcomes.

[51] Leaverton A., Lopes V., Vohr B., Dailey T., Phipps M.G., Allen R.H. (2016). Postpartum contraception needs of women with preterm infants in the neonatal intensive care unit. J. Perinatol..

[52] Ludwig R.J., Austin J., Woodward L.J., Adams L.C., Jaffe M.E., Myers M.M., Welch M.G. (2025). Development and validation of the Mother’s Socioemotional Support Circle (MSSC): A self-evaluation social support tool to guide social support efforts for preterm mothers in the NICU. Acta Psychol..

[53] Alves E., Amorim M., Nogueira C., Silva S. (2023). Quality of Life of Mothers and Fathers 4 to 6 Months After Birth: The Effect of a Very Preterm Delivery. Matern. Child Health J..

[54] Bonet M., Forcella E., Blondel B., Draper E.S., Agostino R., Cuttini M., Zeitlin J. (2015). Approaches to supporting lactation and breastfeeding for very preterm infants in the NICU: A qualitative study in three European regions. BMJ Open.

[55] Coffman S., Levitt M.J., Deets C. (1991). Personal and professional support for mothers of NICU and healthy newborns. J. Obstet. Gynecol. Neonatal Nurs..

[56] Eduku S., Annan E., Amponsah M.A. (2024). Maternal social support and resilience in caring for preterm newborns at the neonatal intensive care unit (NICU): A qualitative study. Heliyon.

[57] Gonçalves J.L., Fuertes M., Alves M.J., Antunes S., Almeida A.R., Casimiro R., Santos M. (2020). Maternal pre and perinatal experiences with their full-term, preterm and very preterm newborns. BMC Pregnancy Childbirth.

[58] Dutra Brazão Lelis B., Isicawa de Sousa M., Faleiros de Mello D., Wernet M., Ferreira Velozo A.B., Moraes Leite A. (2018). Maternal reception in the context of prematurity. J. Nurs. UFPE Rev. Enferm. UFPE.

[59] Lilliesköld S., Zwedberg S., Linnér A., Jonas W. (2022). Parents’ Experiences of Immediate Skin-to-Skin Contact After the Birth of Their Very Preterm Neonates. J. Obstet. Gynecol. Neonatal Nurs..

[60] McGowan E.C., Du N., Hawes K., Tucker R., O’Donnell M., Vohr B. (2017). Maternal Mental Health and Neonatal Intensive Care Unit Discharge Readiness in Mothers of Preterm Infants. J. Pediatr..

[61] Roller C.G. (2005). Getting to know you: Mothers’ experiences of kangaroo care. J. Obstet. Gynecol. Neonatal Nurs..

[62] Scholten N., Mause L., Horenkamp-Sonntag D., Klein M., Dresbach T. (2022). Initiation of lactation and the provision of human milk to preterm infants in German neonatal intensive care units from the mothers’ perspective. BMC Pregnancy Childbirth.

[63] Yu Y., Liu Q., Xiong X., Luo Y., Xie W., Song W., Fu M., Yang Q., Yu G. (2023). Breastfeeding needs of mothers of preterm infants in China: A qualitative study informed by the behaviour change wheel. Int. Breastfeed. J..

[64] Yue J., Liu J., Williams S., Zhang B., Zhao Y., Zhang Q., Zhang L., Liu X., Wall S., Wetzel G. (2020). Barriers and facilitators of kangaroo mother care adoption in five Chinese hospitals: A qualitative study. BMC Public Health.

[65] Abukari A.S., Agyeibi E., Adobea I., Haruna I.S., Korsah E.K. (2025). Navigating preterm motherhood: Perceived support and coping strategies in neonatal intensive care units. J. Neonatal Nurs..

[66] Adu-Bonsaffoh K., Tamma E., Nwameme A.U., Mocking M., Osman K.A., Browne J.L. (2022). Women’s lived experiences of preterm birth and neonatal care for premature infants at a tertiary hospital in Ghana: A qualitative study. PLoS Glob. Public Health.

[67] Alghamdi A.A., Althekrallah A.Y., Sulayil F.A.M., Al Shawan D.S. (2025). Mother’s lived experiences of preterm birth. Int. J. Afr. Nurs. Sci..

[68] Amankwaa L.C., Pickler R.H., Boonmee J. (2007). Maternal responsiveness in mothers of preterm infants. Newborn Infant Nurs. Rev..

[69] Brockway M., Benzies K., Carr E., Aziz K. (2020). Does breastfeeding self-efficacy theory apply to mothers of moderate and late preterm infants? A qualitative exploration. Breastfeeding self-efficacy. J. Clin. Nurs..

[70] Chang Lee S., Long A., Boore J. (2009). Taiwanese women’s experiences of becoming a mother to a very-low-birth-weight preterm infant: A grounded theory study. Int. J. Nurs. Stud..

[71] Chen M.J., Kair L.R., Schwarz E.B., Toland M., Rizzo J., Creinin M.D. (2025). Postpartum Care Recommendations from Parents of Premature Infants Requiring Intensive Care. Matern. Child Health J..

[72] Daliri D.B., Laari L., Ayine A.A., Dei-Asamoa R., Volematome B.G., Bogee G., Apo-Era M.A., Oppong S.A., Abagye N., Jarbaab M. (2024). Psychosocial experiences of mothers of preterm babies admitted to the neonatal intensive care unit of the Upper East Regional Hospital, Bolgatanga: A descriptive phenomenological study. BMJ Open.

[73] Fowler C., Green J., Elliott D., Petty J., Whiting L. (2019). The forgotten mothers of extremely preterm babies: A qualitative study. J. Clin. Nurs..

[74] Gianni M.L., Bezze E., Sannino P., Baro M., Roggero P., Muscolo S., Plevani L., Mosca F. (2018). Maternal views on facilitators of and barriers to breastfeeding preterm infants. BMC Pediatr..

[75] Goutaudier N., Lopez A., Séjourné N., Denis A., Chabrol H. (2011). Premature birth: Subjective and psychological experiences in the first weeks following childbirth, a mixed-methods study. J. Reprod. Infant Psychol..

[76] Herzberg J., Thierfelder A., Ewers M. (2016). Unterstützungsbedürfnisse von Müttern in der neonatologischen Intensivversorgung nach einer Frühgeburt. Klin. Pflegeforsch..

[77] Park J., Bang K.-S. (2022). The physical and emotional health of South Korean mothers of preterm infants in the early postpartum period: A descriptive correlational study. Child Health Nurs. Res..

[78] Palmquist A.E.L., Holdren S., Fair C.D. (2020). “It was all taken away”: Lactation, embodiment, and resistance among mothers caring for their very-low-birth-weight infants in the neonatal intensive care unit. Soc. Sci. Med..

[79] Witt R.E., Colvin B.N., Lenze S.N., Forbes E.S., Parker M.G.K., Hwang S.S., Rogers C.E., Colson E.R. (2022). Lived experiences of stress of Black and Hispanic mothers during hospitalization of preterm infants in neonatal intensive care units. J. Perinatol..

[80] Woodward L.J., Bora S., Clark C.A., Montgomery-Hönger A., Pritchard V.E., Spencer C., Austin N.C. (2014). Very preterm birth: Maternal experiences of the neonatal intensive care environment. J. Perinatol..

[81] Ionio C., Colombo C., Brazzoduro V., Mascheroni E., Confalonieri E., Castoldi F., Lista G. (2016). Mothers and Fathers in NICU: The Impact of Preterm Birth on Parental Distress. Eur. J. Psychol..

[82] Treyvaud K., Lee K.J., Doyle L.W., Anderson P.J. (2014). Very preterm birth influences parental mental health and family outcomes seven years after birth. J. Pediatr..

[83] Vigod S.N., Villegas L., Dennis C.L., Ross L.E. (2010). Prevalence and risk factors for postpartum depression among women with preterm and low-birth-weight infants: A systematic review. BJOG.

[84] Brandon D.H., Tully K.P., Silva S.G., William F.M., Murtha A.P., Turner B.S., Holditch-Davis D. (2011). Emotional responses of mothers of late-preterm and term infants. J. Obstet. Gynecol. Neonatal Nurs..

[85] Flacking R., Lehtonen L., Thomson G., Axelin A., Ahlqvist S., Moran V.H., Ewald U., Dykes F. (2012). Seperation and Closeness Experiences in the Neonatal Enviroment (SCENE) Group. Closeness and separation in neonatal intensive care. Acta Paediatr..

[86] Donabedian A. (1988). The quality of care: How can it be assessed?. JAMA.

